# Connexin 43 and Pannexin 1 in Renal Cell Populations in Diabetic Kidney Disease

**DOI:** 10.3390/ijms27052152

**Published:** 2026-02-25

**Authors:** Marinela Jelinčić Korčulanin, Anita Racetin, Nikola Pavlović, Ivo Jeličić, Merica Glavina Durdov, Monika Andrzejewska, Leo Jerčić, Ivana Bočina, Nives Kević, Ivana Restović, Katarina Vukojević, Patricija Bajt, Karla Svaguša, Natalija Filipović

**Affiliations:** 1 Department of Anatomy, Histology and Embryology, University of Split School of Medicine, 21000 Split, Croatia; marinela.jelincic.korculanin@mefst.hr (M.J.K.); anita.racetin@mefst.hr (A.R.); nikola.pavlovic@mefst.hr (N.P.); monika.andrzejewska@uwm.edu.pl (M.A.); leo.jercic@gmail.com (L.J.); kvukojev@gmail.com (K.V.); patricija.bajt@mefst.hr (P.B.); karla.svagusa@mefst.hr (K.S.); 2 Renal Unit, Clinical Hospital of Split, 21000 Split, Croatia; ivojelicic@gmail.com; 3 Department of Pathology, University Hospital of Split, 21000 Split, Croatia; merigdst@yahoo.co.uk; 4 Department of Animal Anatomy and Physiology, Faculty of Biology and Biotechnology, University of Warmia and Mazury in Olsztyn, 10-719 Olsztyn, Poland; 5 Department of Biology, Faculty of Science, University of Split, 21000 Split, Croatia; bocina@pmfst.hr (I.B.); nkevic@pmfst.hr (N.K.); 6 Department of Teacher Education, University of Split Faculty of Humanities and Social Sciences, 21000 Split, Croatia; irestovic@ffst.hr

**Keywords:** connexin 43, pannexin 1, diabetic nephropathy, cell populations

## Abstract

We studied the expression of connexin 43 (Cx43) and pannexin 1 (PANX1) in different cellular populations of the kidneys of diabetic mice and diabetic and non-diabetic patients, to evaluate their role as potential therapeutic targets in diabetic kidney disease (DKD). A combination of a low dose of streptozotocin and a high-fat diet (HFD) was used to induce a type 2 diabetes model (DM2) in mice. Kidney tissues from diabetic (*n* = 9) and control patients (*n* = 11) who underwent nephrectomy were collected. Tissues from mice and humans were processed for double immunofluorescence, using antibodies against Cx43, phosphorylated Cx43 (pCx43) or PANX1 and markers for specific cell populations: endothelium (CD31/PECAM1); pericytes/mesangium (PDGFRB); podocytes (nephrin/synaptopodin); proximal tubules and collecting ducts (aquaporin 2). The results showed a significant decrease in the expression of pCx43 in PDGFRB-immunoreactive mesangium in diabetic patients compared to the control group (*p* < 0.0001). This contrasted with an increase in pCx43 in pericytes of diabetic mice (*p* = 0.1). However, we found a general decrease in Cx43 protein expression in diabetic mouse kidneys (*p* < 0.05). We also found a decrease in the expression of PANX1 in endothelial cells of diabetic patients (*p* < 0.05) and a significant increase in PANX1 expression in cells expressing PDGFRB (*p* < 0.05). Expression of PANX1 in endothelium (r = −0.50; *p* < 0.05) and pCx43 in the mesangium (r = −0.65; *p* < 0.01) correlated negatively with the percentage of sclerotic glomeruli. The expression and activation of Cx43 and the expression of PANX1 are altered in distinct populations of renal cells during long-term type 2 diabetes mellitus, especially cells of the vascular wall. This may indicate their role in the pathophysiological processes of DKD. Therefore, connexin and pannexin channels could be considered as possible therapeutic targets in the prevention and treatment of diabetic kidney disease.

## 1. Introduction

The kidneys are organs whose function is essential for the regulation of body water, electrolyte and acid-base balance. Diabetic kidney disease (DKD) is currently the most common cause of chronic kidney disease and end-stage renal failure worldwide [1]. DKD develops as a result of complex interactions between metabolic, hemodynamic, inflammatory, oxidative, and profibrotic cascades, which lead to progressive damage to the glomerular and tubulointerstitial compartments [2]. Chronic hyperglycemia initiates a cascade of molecular mechanisms involving the accumulation of advanced glycation end products (AGEs), activation of the polyol pathway and protein kinase C (PKC), mitochondrial dysfunction and increased oxidative stress, which together damage glomerular and tubular cells [2]. At the same time, there is an increase in intraglomerular pressure, activation of the renin–angiotensin–aldosterone axis, endothelial dysfunction and development of inflammation involving cytokines, chemokines, and immune cells [3,4]. Histologically, DKD is characterized by thickening of the glomerular basement membrane, diffuse and nodular mesangial sclerosis, podocyte loss, arteriolar sclerosis, progressive interstitial fibrosis, and tubular atrophy [2]. In recent decades, there has been growing interest in the role of intercellular communication and membrane channels in the pathogenesis of DKD, especially proteins from the connexin and pannexin families [5,6,7,8]. Connexins are transmembrane proteins that form gap junctions (GJ) between neighbouring cells and hemichannels in the cell membrane, allowing the transfer of small molecules and ions and enabling the exchange of ions, metabolites, and small-molecule signalling. The connexin family includes more than 20 isoforms (21 in humans), nine of which are expressed in the kidneys [9,10,11], with Cx40, Cx43, and Cx45 being the major isoforms [12]. Connexin 43 (Cx43) is the best-known connexin in the kidney [13]. Cx43 regulates proliferation, differentiation, inflammatory response, and fibrogenesis, and its expression is altered in many kidney diseases, including acute and chronic models of nephropathy [14]. In the human kidney, Cx43 has been detected in podocytes, mesangial cells, tubular epithelium, vascular endothelium, and smooth muscle. In various forms of chronic kidney disease, Cx43 has been found to be overexpressed and/or deregulated in the glomeruli and interstitium [15]. It has been shown that during diabetic nephropathy, Cx43 in podocytes undergoes a change in distribution, which correlates with the severity of damage to these cells and a poorer renal prognosis. In addition, Cx43 expression in the tubular epithelium is associated with epithelial–mesenchymal transition (EMT), extracellular matrix deposition, and interstitial fibrosis [16]. Studies on mouse models have also shown that modulation of Cx43—both its inhibition in the context of proinflammatory overexpression and restoration of normal levels in tubular cells—can protect against the progression of chronic kidney damage and fibrosis [17].

Pannexin 1 (PANX1) is a membrane glycoprotein that forms hexameric channels, which, upon activation, allow ATP and other small molecules to flow into the extracellular space, serving as a source of damage-associated molecular patterns (DAMPs) and an important component of the purinergic signalling pathway [8]. In the kidney, PANX1 is widely expressed in the nephron epithelium—particularly in the proximal tubules, thin limbs of the loop of Henle, distal tubule, renin-producing cells, and the wall of afferent arterioles—where it regulates ATP release, modulating tubular transport, inflammatory response, and renin–angiotensin–aldosterone system activity [18,19]. Experimental studies have shown that PANX1 hyperactivity in proximal tubule cells and mesangium promotes mitochondrial dysfunction, increases cell death, and activates the inflammasome, while pharmacological or genetic inhibition of PANX1 can limit kidney damage in models of acute and chronic nephropathy [20,21].

Both Cx43 and PANX1 play a key role in regulating ATP release and purinergic signal propagation in the kidney, which is particularly important in the environment of chronic hyperglycemia, oxidative stress, and inflammation characteristic of DKD [11]. Experimental data suggest the existence of a Cx43/PANX1–ATP–purinergic receptor (including P2X7) axis, which may create a positive feedback loop that intensifies inflammatory reactions, fibrosis, and damage to podocytes and tubular cells during the course of diabetic nephropathy [22].

Despite numerous studies highlighting the link between disrupted connexin and ATP signalling-mediated intercellular communication in diabetic kidney disease, gaps in our knowledge remain, including incomplete agreement on which cell types express individual connexin and pannexin isoforms. In this context, quantitative and qualitative assessment of Cx43 and PANX1 expression in different kidney cell populations of diabetic patients is an important step toward understanding the pathophysiological mechanisms of DKD and may identify new targets for nephroprotective therapy [11].

## 2. Results

### 2.1. Type 2 Diabetes Model in Mice

**Figure 1:**
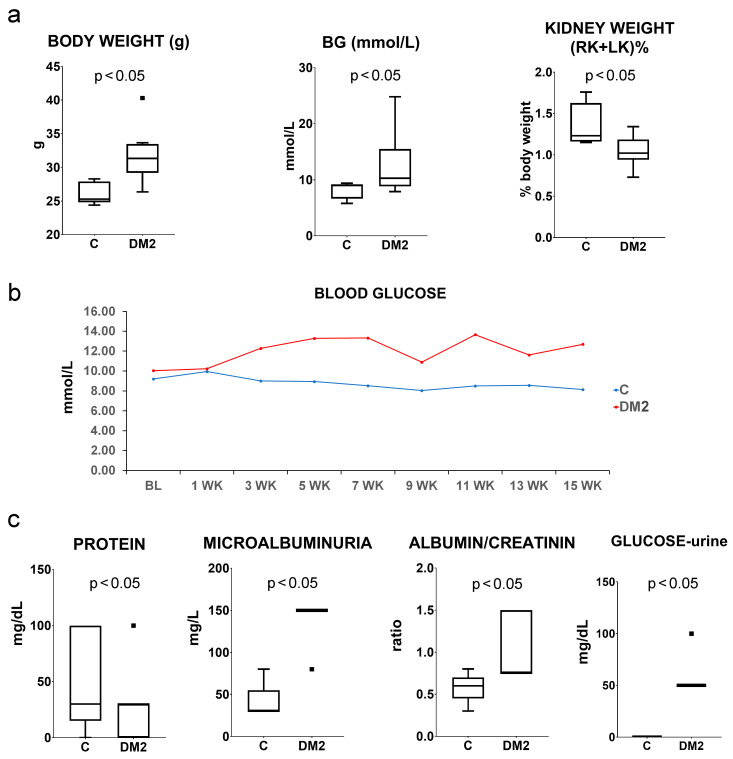
Body weight, blood glucose, kidney weight, and urine parameters in control (C) and diabetic mice (DM2). (**a**) Body weight; BG—blood glucose at the 15th week of the experiment; kidney weight (RK—right kidney; LK—left kidney) expressed as a percentage of body weight. (**b**) Mean blood glucose in C and DM2 groups of mice during the experiment. (**c**) Microalbuminuria, albumin/creatinine ratio, and glucose in the urine of experimental mice. Tukey plots; *p* < 0.05 indicates a statistically significant difference between the control and diabetic groups (*t*-test for unequal variances).

A combination of streptozotocin and a high-fat diet (HFD) was used to induce a type 2 diabetes model (DM2) in mice. The experiment lasted for 15 weeks. The results showed significantly higher (*p* < 0.05) body mass in the DM2 group compared to the control group of mice (C). Diabetic mice had significantly higher glucose levels (*p* < 0.05), while their renal weight, expressed as a percentage of body mass, was significantly lower (*p* < 0.05; Figure 1a). Mean blood glucose remained persistently higher in the DM2 group from the third week until the end of the experiment (Figure 1b). In the urine of diabetic mice, we found significantly higher (*p* < 0.05) microalbuminuria, albumin/creatinine ratio, and glucose (Figure 1c).

**Figure 2:**
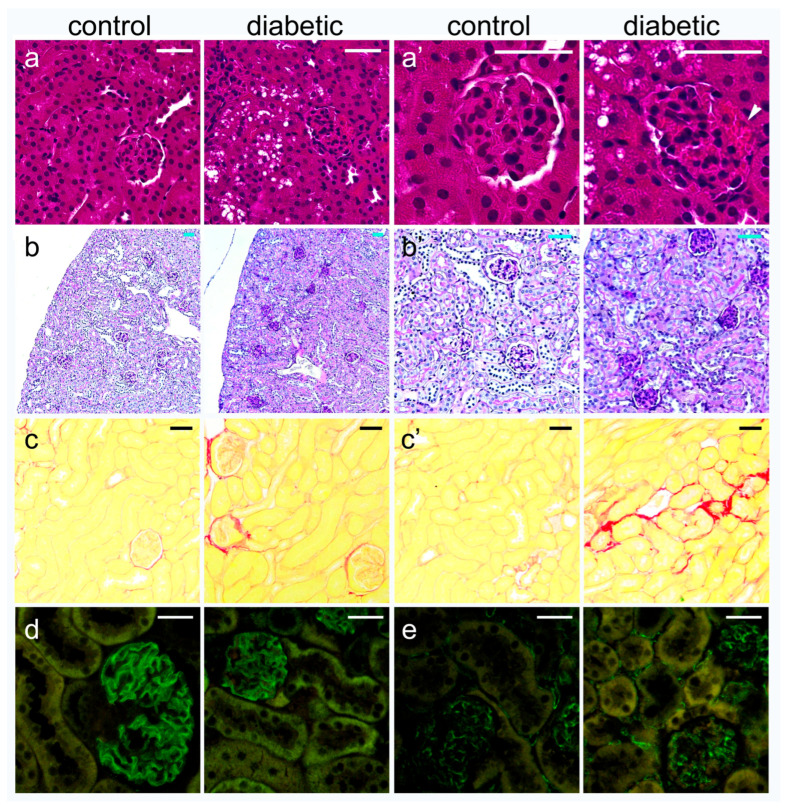
Representative photomicrographs of histological staining and immunohistochemical markers of renal damage in experimental mice. (**a**,**a’**) Hematoxylin-eosin (HE) staining; (**a’**) is a magnification of (**a**). (**b**,**b’**) Periodic acid Schiff staining (PAS); (**b’**) is a magnification of (**b**). (**c**,**c’**) Sirius red staining; (**c**)—glomerular sclerosis, (**c’**)—tubulointerstitial sclerosis in diabetic mice. (**d**) Immunofluorescent staining using anti-podocin antibodies (podocin, green). (**e**) Immunofluorescent staining using anti-CD31 antibodies (CD31, green). Scale bars = 50 µm.

**Figure 3:**
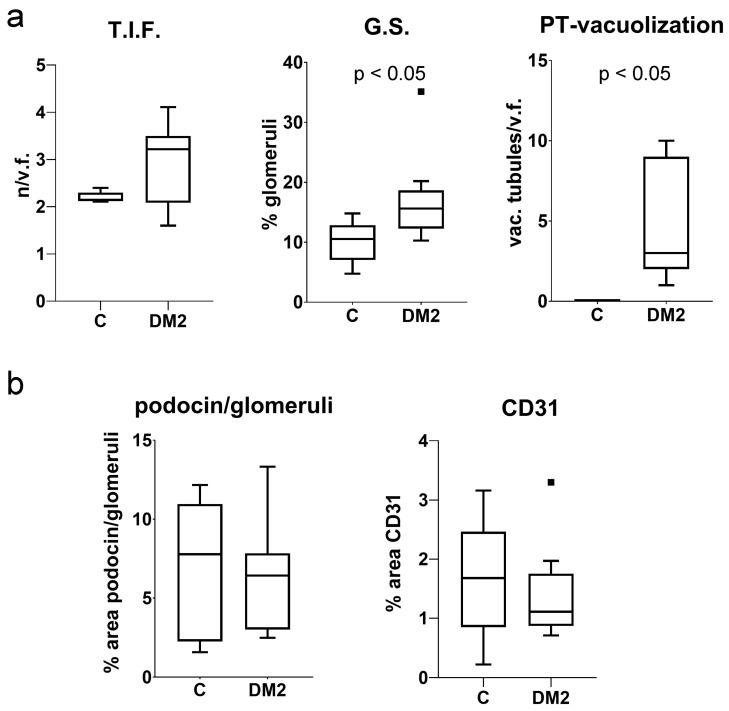
Measurement of kidney damage in experimental mice. (**a**) T.I.F.—tubulointerstitial fibrosis, expressed as the number of T.I.F. foci per visual field (n/v.f.); G.S.—glomerular sclerosis, expressed as the percentage of glomeruli positive for collagen in Sirius red staining; PT-vacuolization—percentage of proximal tubules with cytoplasmic vacuoles (lipid droplets) on HE staining. (**b**) Results of the analysis of immunofluorescent staining for podocin, expressed as the positive percentage area of the glomeruli and CD31, expressed as the positive percentage area of the analyzed section. Tukey plots; *p* < 0.05—statistically significant difference between the control and diabetic groups (*t*-test for unequal variances).

Pathohistological examination revealed a dense structure of the glomeruli (Figure 2a,b) and vacuolization of the proximal tubule (Figure 2a,a’). In addition, sclerotic changes in the glomeruli and tubulointerstitial sclerosis (Figure 2c,c’) were observed. Expression of podocin (Figure 2d) and endothelial marker CD31 (Figure 2e) was not obviously changed. The analysis did not confirm significant differences in the number of tubulointerstitial fibrotic foci (T.I.F.) or in the immunohistochemical expression of podocin and CD31 (*p* > 0.05; Figure 3a,b). On the other hand, the DM2 group had a significantly higher percentage of sclerotic glomeruli and vacuolated proximal tubules (*p* < 0.05; Figure 3a). Vacuolated tubules were not observed in the control group (Figure 2a and Figure 3a).

**Figure 4:**
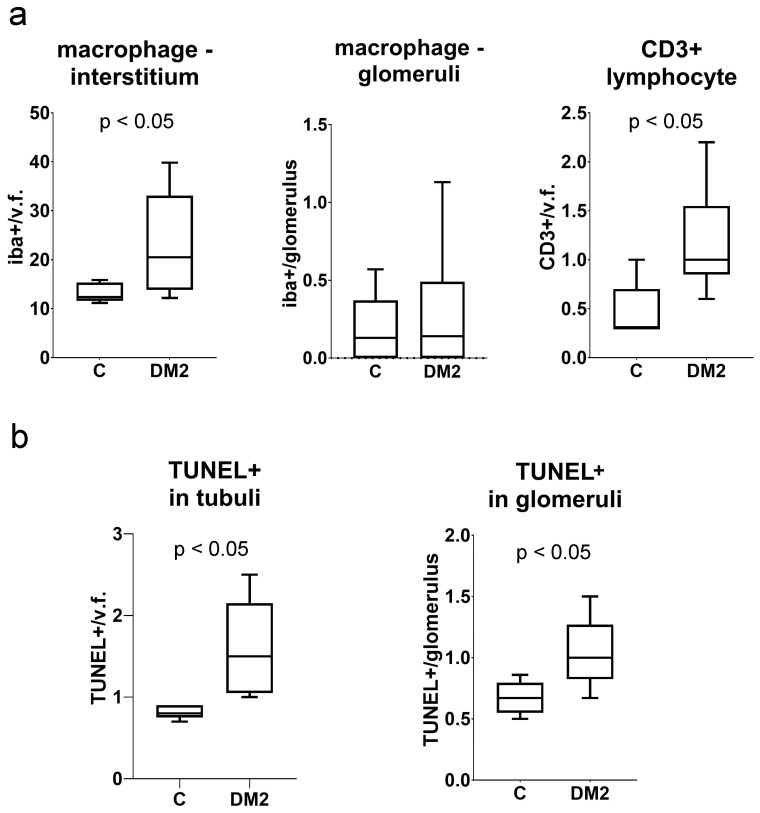
Measurement of leukocyte infiltration and apoptosis in the kidneys of experimental mice. (**a**) Macrophage infiltration, expressed as the number of Iba1-immunoreactive cells in interstitium per visual field (Iba+/v.f.), as well as the number of Iba+ cells per glomerulus; lymphocyte infiltration, expressed as the number of CD3+ cells per visual field (CD3+/v.f.). (**b**) Results of TUNEL staining analysis expressed as the number of TUNEL-positive cells in tubules per visual field (TUNEL+/v.f.) and as TUNEL-positive cells per glomeruli. Tukey plots; *p* < 0.05 indicates a statistically significant difference between the control and diabetic groups (*t*-test for unequal variances).

Macrophage infiltration was not significantly increased in the tubulointerstitial renal compartment of DM2 mice (*p* < 0.05), nor in the glomeruli (*p* > 0.05; Figure 4). There were more CD3-immunoreactive cells in kidney sections from diabetic mice (*p* < 0.05; Figure 4). In addition, we found more TUNEL+ cells in both the glomeruli and the tubulointerstitial compartment of diabetic mice compared to controls (*p* < 0.05; Figure 4b).

**Figure 5:**
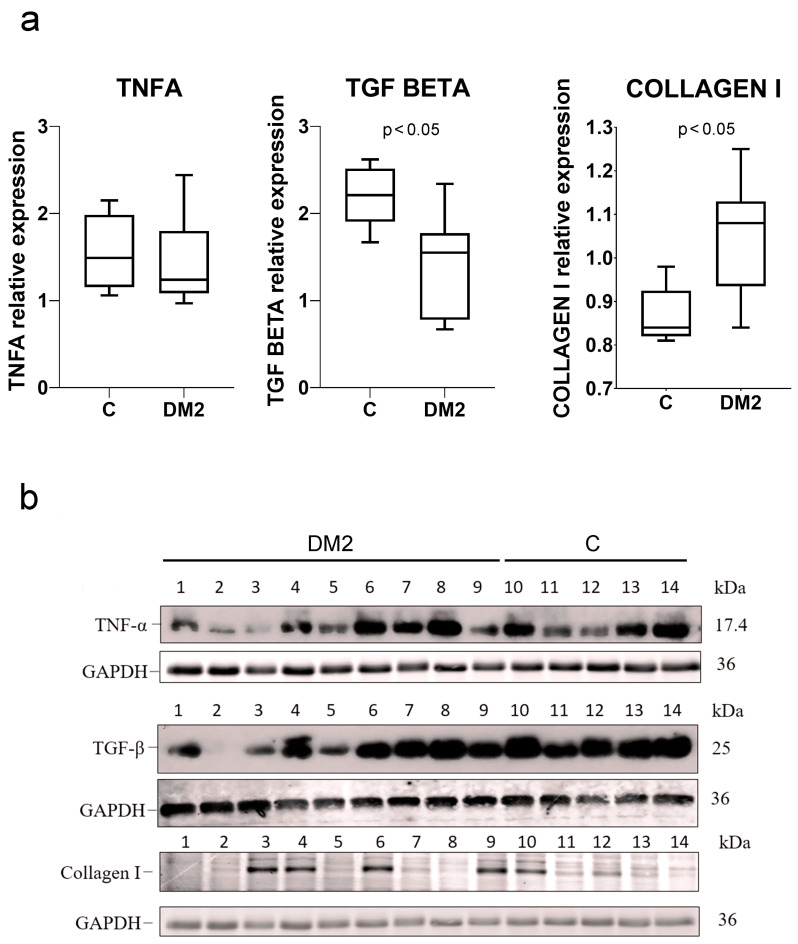
Western blot analysis of kidneys from diabetic and non-diabetic mice. (**a**) Western blot quantification results. Levels of TNF alpha (TNFA), TGF beta, and type I collagen were normalized to GAPDH as an internal loading control. Tukey plots; *p* < 0.05 indicates a statistically significant difference between the control and diabetic groups (*t*-test for unequal variances). (**b**) Immunoblots of kidneys from diabetic and non-diabetic mice.

Protein expression of TNFA did not significantly differ between control and diabetic groups of mice (*p* > 0.05; Figure 5a,b). Expression of TGFB was significantly lower in the DM2 group, while expression of type I collagen was significantly increased in DM2 compared to the control group of mice (*p* < 0.05; Figure 5a,b).

**Figure 6:**
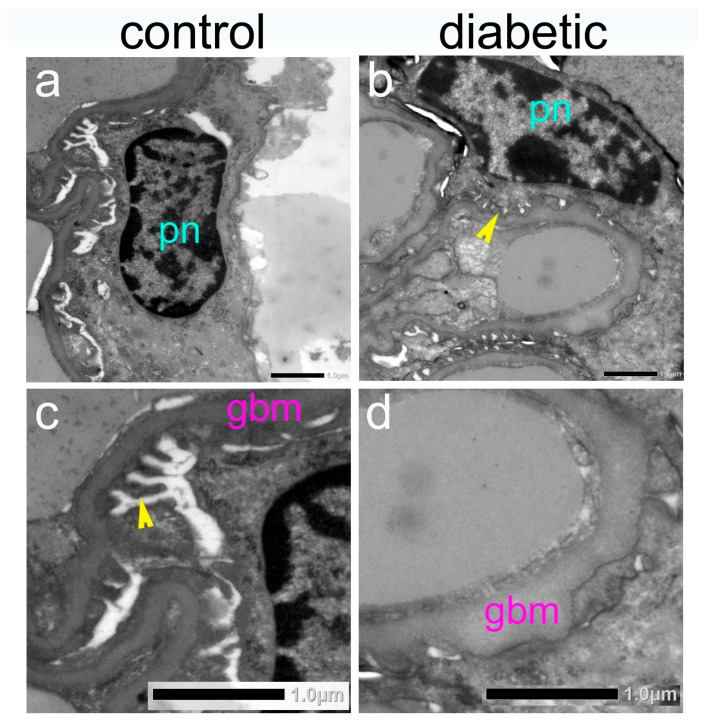
Representative photomicrographs of kidneys from control and diabetic mice using transmission electron microscopy (TEM). (**c**) is a magnified detail from (**a**), and (**d**) is a magnified detail from (**b**). pn—podocyte nucleus; gbm—glomerular basement membrane; arrowheads indicate podocyte pedicles. Thickening of the gbm is evident in diabetic mice. Scale bar = 1.0 µm.

Ultrastructural examination revealed thickening of the glomerular basement membrane (Figure 6) and confirmed the presence of lipid droplets in the epithelium of the proximal tubules of diabetic mice.

### 2.2. Expression of Connexin 43 in Mouse Kidneys

**Figure 7:**
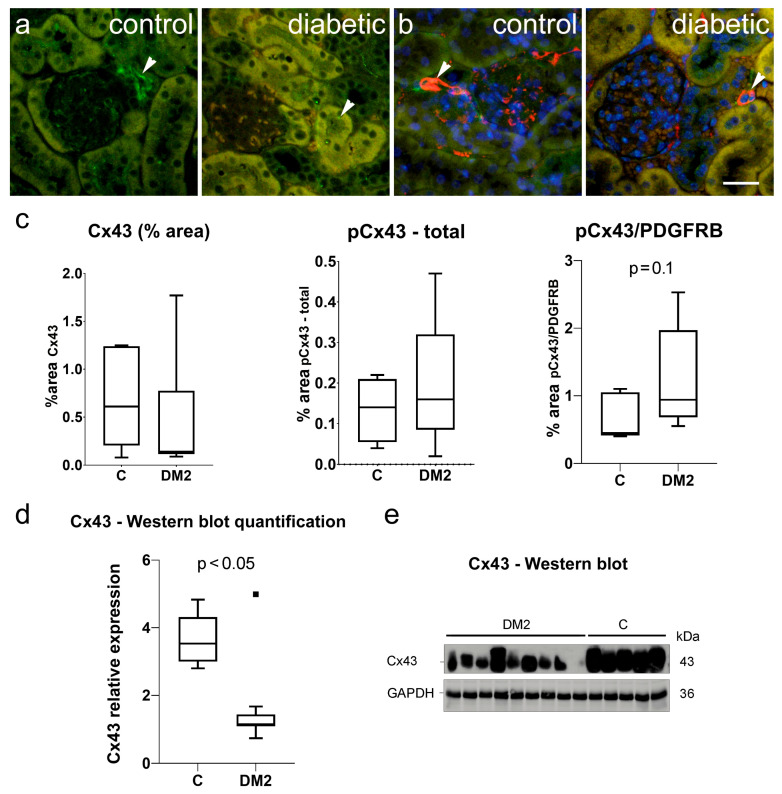
Connexin 43 (Cx43) expression in kidneys of control and diabetic mice. (**a**) Representative photomicrographs of Cx43 (Cx43, green) expression in the kidneys of control and diabetic mice; (**b**) Representative photomicrographs of phosphorylatedCx43 (pCx43, green) expression in the kidneys of control and diabetic mice, co-localized with PDGFRB, a pericyte marker (PDGFRB, red); co-localization appears yellow). Nuclei in panel b are stained blue. Arrowheads indicate positive Cx43/pCx43 staining. (**c**) Results of the analysis of staining for Cx43, total pCx43, and pCx43 co-localized with PDGFRB, expressed as percentage area. (**d**) Western blot analysis of Cx43 in the kidneys of diabetic and non-diabetic mice. The amount of Cx43 was normalized to GAPDH as an internal control. Tukey plots; *p* < 0.05 indicates a statistically significant difference between control and diabetic groups (*t*-test for unequal variances). (**e**) Immunoblots of kidneys from diabetic and non-diabetic mice. Scale bar = 50 µm (refers to all).

Immunohistochemical expression of Cx43 and pCx43 was observed in sections from a mouse kidney as rare green antibody granular formations (Figure 7a,b). In the case of pCx43, these partially colocalized with the pericyte marker—PDGFRB (Figure 7b). Immunohistochemically, we did not observe a significant difference in Cx43 expression between diabetic and control mice, although it tended to be lower in the DM2 group (*p* > 0.05; Figure 7b). The expression of the activated form, pCx43, also did not differ between groups when the total amount was measured (*p* > 0.05; Figure 7b). However, when only pCx43 colocalized with PDGFRB was considered, increased expression was observed at the level of *p* = 0.1. Protein analysis using Western blot revealed a highly and significantly decreased expression in total Cx43 in diabetic mice compared to the control group (*p* < 0.05).

### 2.3. Expression of Pannexin 1 in Mouse Kidneys

**Figure 8:**
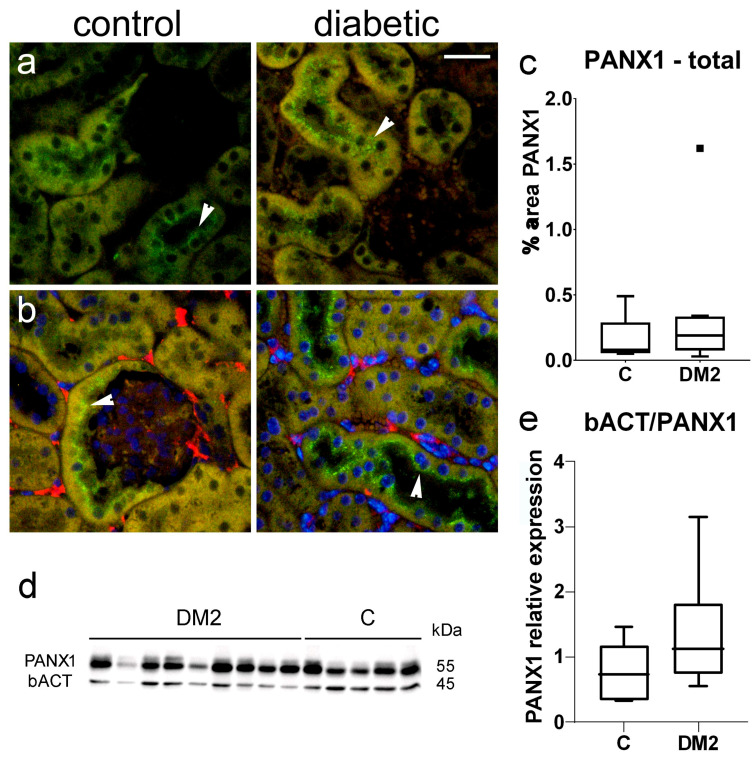
Pannexin 1 (PANX1) expression in the kidneys of control and diabetic mice. (**a**) Representative photomicrographs showing PANX1 (PANX1, green) expression in the kidneys of control and diabetic mice; (**b**) Representative photomicrographs showing PANX1 (green) expression in the kidneys of control and diabetic mice, co-localized with PDGFRB, a pericyte marker (PDGFRB, red). Nuclei in panel (**b**) are stained blue. Arrowheads indicate positive PANX1 staining. (**c**) Results of the analysis of PANX1 staining in renal tissue sections, expressed as percentage area. Tukey plots; no significant difference (*p* > 0.05) was found between the control and diabetic groups (*t*-test for unequal variances). Scale bar = 50 µm (refers to all). (**d**) Immunoblots of kidneys from diabetic and non-diabetic mice. (**e**) Western blot analysis of PANX1 in the kidneys of diabetic and non-diabetic mice. The amount of PANX1 was normalized to beta actin as an internal control. Tukey plots; *p* < 0.05 indicates a statistically significant difference between control and diabetic groups (*t*-test for unequal variances).

Immunohistochemical expression of PANX1 was observed in sections of mouse kidney as green, granular antibody formations (Figure 8a,b), which partially colocalized with pericyte marker PDGFRB (Figure 8b). Immunohistochemically, we did not observe a significant difference in PANX1 expression between diabetic and control mice (*p* > 0.05; Figure 8c).

### 2.4. Expression of Connexin 43 in Different Cellular Populations of Human Diabetic and Non-Diabetic Kidneys

**Figure 9:**
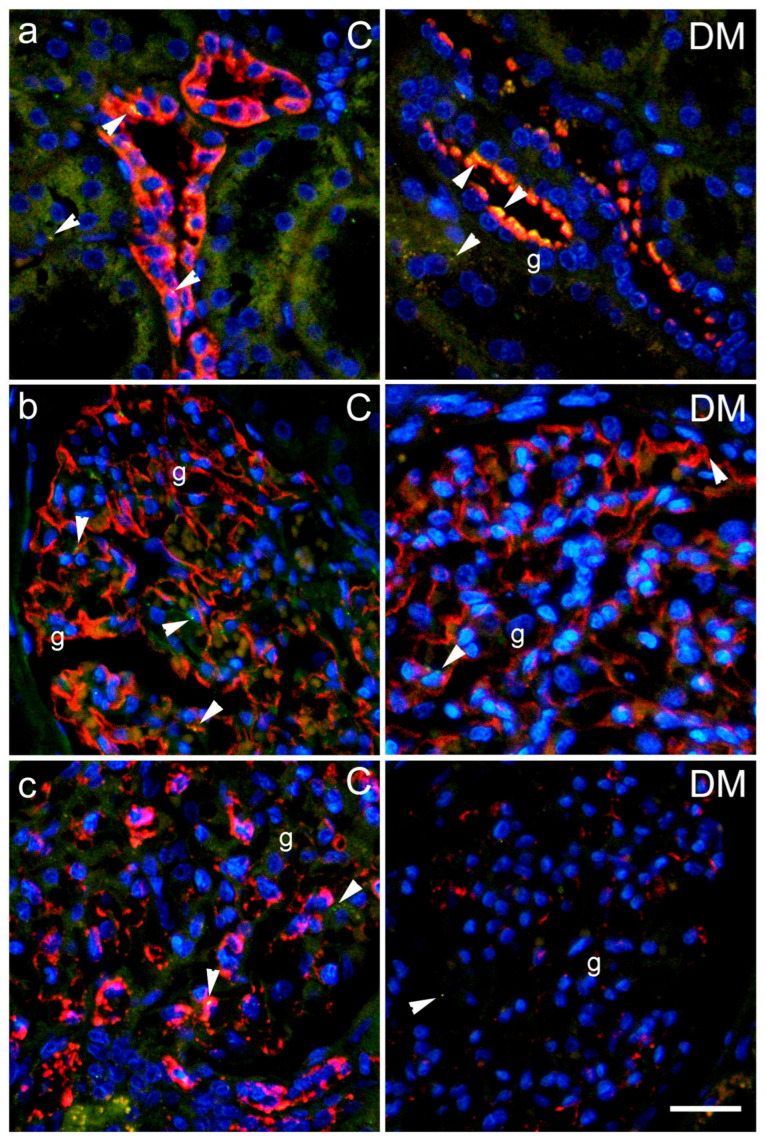
Connexin 43 (Cx43) expression in different cellular populations of renal tissue sections from control (C) and diabetic patients (DM). Representative photomicrographs show Cx43 (Cx43, green) expression in the kidneys of control and diabetic groups co-localized with (**a**) aquaporin 2 (AQP2, red), a marker for collecting duct epithelium; (**b**) synaptopodin, a marker for podocytes (synaptopodin, red) and (**c**) PDGFRB, a marker for pericytes and mesangial cells (PDGFRB, red). g—glomerulus; arrowheads indicate positive Cx43 staining. Nuclei are stained blue. Scale bar = 50 µm (refers to all).

**Figure 10:**
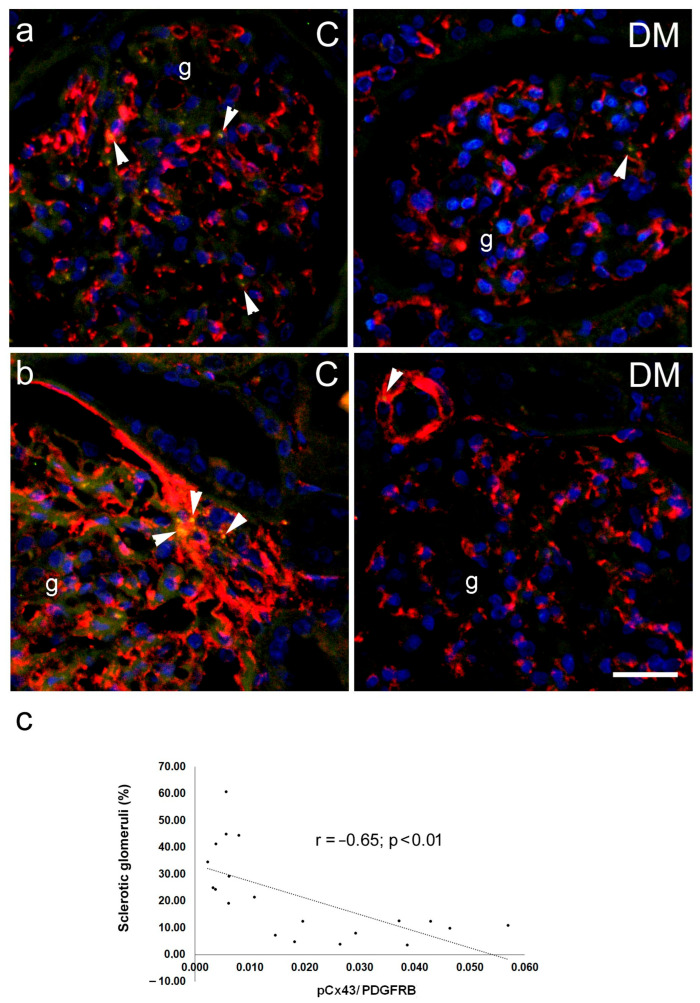
Connexin 43 (Cx43) expression in different cellular populations of renal tissue sections from control (C) and diabetic patients (DM). (**a**) Representative photomicrographs of Cx43 (Cx43, green) expression in kidneys of control and diabetic groups co-localized with CD31 (CD31, red), a marker for endothelial cells. (**b**) Representative photomicrographs of phospho-Cx43 (PcX43, green) expression co-localized with PDGFRB, a marker for pericytes and mesangial cells (PDGFRB, red). g—glomerulus; arrowheads indicate positive Cx43 staining. Nuclei are stained blue. (**c**) Pearson’s correlation between expression of pCx43 in the mesangium (pCx43/PDGFRB) and the percentage of sclerotic glomeruli. Scale bar = 50 µm (refers to all).

Immunohistochemical expression of Cx43 and pCx43 was observed in sections from human kidney as rare green antibody granular formations (Figure 9 and Figure 10), which partially colocalized with markers for the collecting duct epithelium aquaporin 2 (AQP2); synaptopodin, a marker for podocytes; PDGFRB, a marker for pericytes and mesangial cells (Figure 9c and Figure 10b) and CD31, a marker for endothelial cells (Figure 10a).

**Figure 11:**
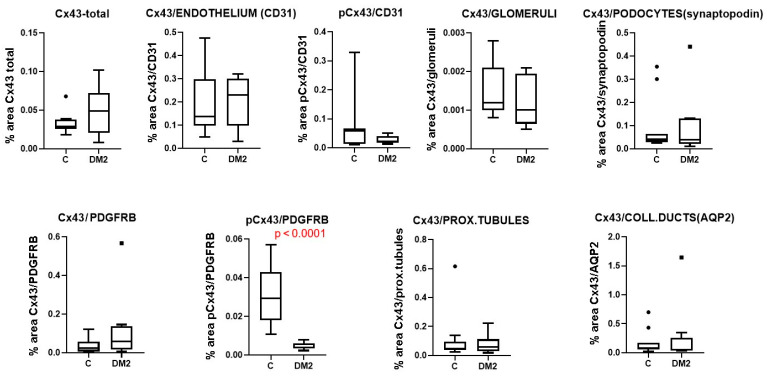
Results of the analysis of staining for connexin 43 (Cx43) and phospho Cx43 (pCx43) in different cellular populations of renal tissue sections from control (C) and diabetic (DM2) patients, expressed as percentage area. Tukey plots; *p* < 0.05 indicates a statistically significant difference between the control and diabetic groups (*t*-test for unequal variances).

Immunohistochemically, we did not observe a significant difference in total Cx43 expression between diabetic and control patients (Figure 11), nor in the expression of Cx43 in glomerular endothelium, podocytes, mesangium, proximal tubule, or collecting ducts epithelium (*p* > 0.05; Figure 11). For pCx43, we also did not find significant differences between groups when the total amount was measured (*p* > 0.05; Figure 11). However, when only pCx43 colocalized with PDGFRB was expressed, a highly significant decrease in expression was found, at the level of *p* < 0.0001 (Figure 11). Moreover, a significant negative correlation was found between the expression of pCx43 in the mesangium (colocalization with PDGFRB) and the percentage of sclerotic glomeruli, measured in Sirius red-stained sections, which served as a measure of renal damage (Pearson’s r = −0.65, *p* < 0.01; Figure 10c).

### 2.5. Expression of Pannexin 1 in Different Cellular Populations of Human Diabetic and Non-Diabetic Kidneys

**Figure 12:**
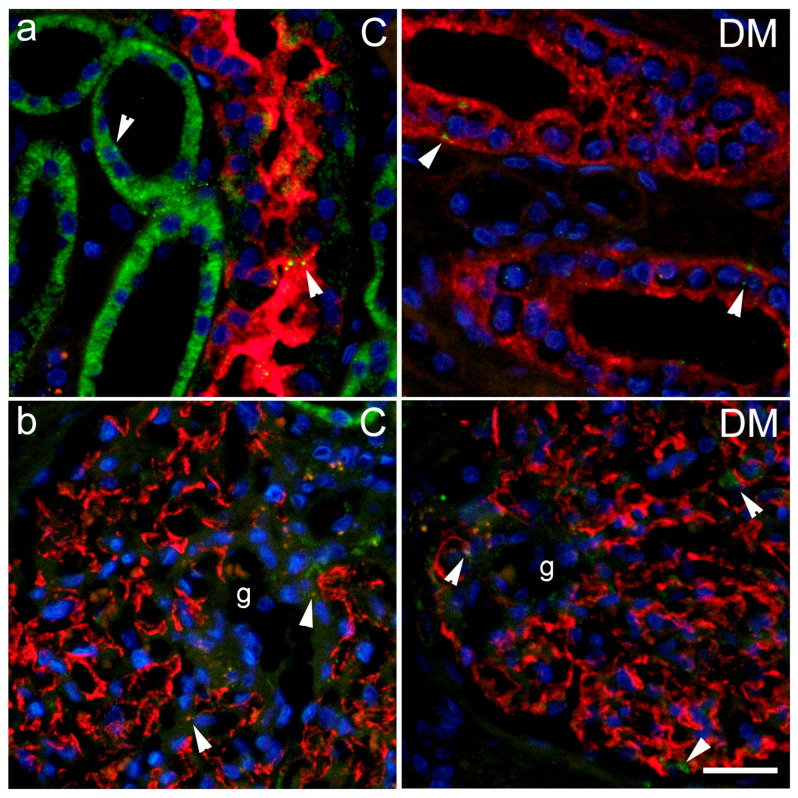
Pannexin 1 (PANX1) expression in different cellular populations of renal tissue sections from control (C) and diabetic (DM) patients. Representative photomicrographs show PANX1 (PANX1, green) expression in the kidneys of control and diabetic groups co-localized with (**a**) aquaporin 2 (AQP2, red), a marker for collecting duct epithelium; (**b**) nephrin (nephrin, red) a marker for podocytes. Arrowheads indicate positive PANX1 staining. Nuclei are stained blue. Scale bar = 50 µm (refers to all). g—glomerulus.

**Figure 13:**
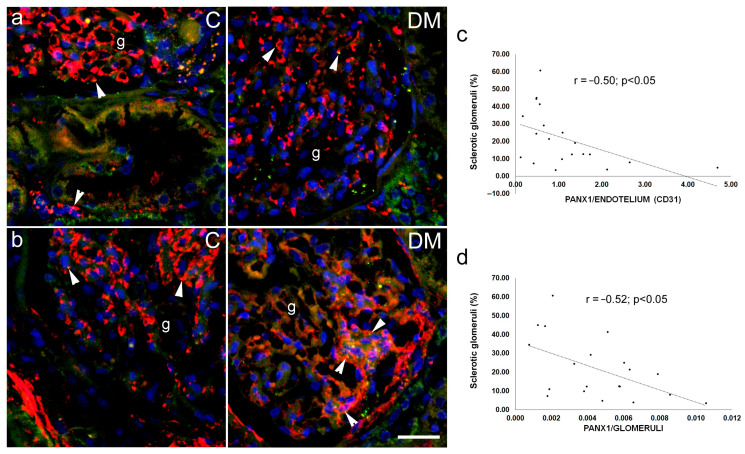
Pannexin 1 (PANX1) expression in different cellular populations of renal tissue sections from control (C) and diabetic (DM) patients. (**a**) Representative photomicrographs show PANX1 (PANX1, green) expression in the kidneys of control and diabetic groups co-localized with CD31 (CD31, red), a marker for endothelial cells. (**b**) Representative photomicrographs show PANX1 (PANX1, green) expression co-localized with PDGFRB (PDGFRB, red), a marker for pericytes and mesangial cells. g—glomerulus; arrowheads indicate positive PANX1 staining. Nuclei are stained blue. (**c**) Pearson’s correlation between PANX1 expression in the endothelium (PANX1/CD31) and percentage of the sclerotic glomeruli. (**d**) Pearson’s correlation between PANX1 expression in the glomeruli and percentage of the sclerotic glomeruli. Scale bar = 50 µm (refers to all).

Immunohistochemical expression of PANX1 was observed in sections from human kidney as green antibody granular formations (Figure 12 and Figure 13), which partially colocalized with markers for the collecting duct epithelium, aquaporin 2 (AQP2; Figure 12a); nephrin, a marker for podocytes (Figure 12b); CD31, a marker for endothelial cells (Figure 13a), and PDGFRB, a marker for pericytes and mesangial cells (Figure 13b).

**Figure 14:**
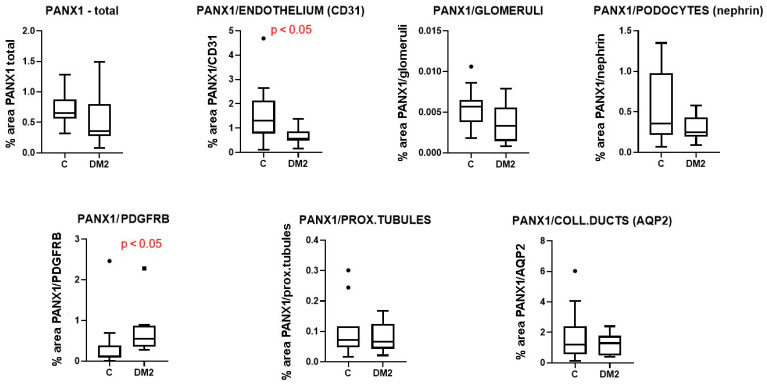
Results of the analysis of staining for Pannexin 1 (PANX1) in different cellular populations of renal tissue sections from control (C) and diabetic patients (DM2), expressed as percentage area. Tukey plots; *p* < 0.05 indicates a statistically significant difference between the control and diabetic groups (*t*-test for unequal variances).

Immunohistochemically, we did not observe a significant difference in total PANX1 expression between diabetic and control patients (Figure 14), nor in PANX1 expression in glomeruli, podocytes, proximal tubules or the collecting duct epithelium (*p* > 0.05; Figure 14). However, PANX1 expression in CD31-immunoreactive endothelium was significantly decreased in diabetic patients compared to control patients (*p* < 0.05; Figure 14). In addition, PANX1 expression in PDGFRB-immunoreactive cells was significantly increased in diabetic patients (*p* < 0.05; Figure 14). Moreover, a significant negative correlation was found between PANX expression 1 in glomerular CD31-immunoreactive endothelium and the percentage of sclerotic glomeruli, which served as a measure of renal damage (Pearson’s r = −0.50, *p* < 0.05; Figure 13c).

## 3. Discussion

We studied the expression of connexin 43 (Cx43) and pannexin 1 (PANX1) in a diabetic mouse model, as well as in different cellular populations of the kidneys from diabetic and non-diabetic patients. A combination of a high dose of streptozotocin and a high-fat diet (HFD) was used to induce a type 2 diabetes model (DM2) in mice, according to Shi et al. [23], and the experiment lasted 15 weeks. The results of the model evaluation confirmed the features of type 2 diabetes, including higher body mass and elevated blood sugar in the diabetic group of mice. Blood glucose concentrations during the experiment were moderately increased, which is consistent with the characteristics of the human disease. Microalbuminuria, a higher albumin/creatinine ratio, and the presence of glucose in the urine of the DM2 group also indicated the success of the diabetes model. The renal weight, expressed as a percentage of body mass, was significantly lower in the DM2 group, which could result from renal atrophy but should mostly be attributed to the higher body mass of diabetic animals. Pathological examination revealed condensation and sclerotic changes in the glomeruli. In addition, strong vacuolization of the proximal tubules was observed in diabetic mice, while vacuoles in the proximal tubules were not found in control mice. In our previous study on a rat model, we demonstrated that the vacuoles observed in the epithelial cells of the proximal tubules in kidneys from diabetic animals are lipid droplets [24]. We also observed lipid droplets in the epithelium of the proximal tubules during this experiment using transmission electron microscopy (TEM). Therefore, we conclude that our diabetic model resulted in substantial metabolic changes in the kidney, particularly in the highly metabolically active epithelium of the proximal tubules, leading to cytoplasmic lipid accumulation. We found increased macrophage infiltration in the tubulointerstitial renal compartment, as well as more CD3-immunoreactive cells, and more TUNEL-positive cells in both the glomeruli and the tubulointerstitial compartment in renal sections of DM2 mice. In addition, type I collagen expression was higher in DM2 mice compared to the control group. A decrease in TGFβ that we found was somewhat unexpected. However, despite TGFβ playing an important role in inflammation and fibrotic processes, its expression profoundly depends on the context [25,26]. Studies have shown that in chronic non-healing lesions, for instance chronic diabetic ulcers, the expression of TGFβ is low, contributing to the prolongation of healing [26]. Ultrastructural examination revealed the thickening of the glomerular basement membrane. However, the observed pathological changes were moderate in magnitude, and we did not find a significantly higher number of tubulointerstitial fibrotic foci. Expression of inflammatory markers and immunohistochemical expression of podocin and CD31 were not significantly different between groups. This could be explained by the fact that, in a chronic diabetic model such as the present one, normal ageing changes in the kidneys of both control and diabetic groups, cannot be avoided, and may mask potential differences between groups.

The network of communication between renal cells during the development of diabetic nephropathy is still not fully understood and remains an intriguing area of research. Therefore, we used immunohistochemistry to study whether the expression of Cx43 and PANX1 is altered in cells of the diabetic kidney.

Immunohistochemically, we did not observe a significant difference in total Cx43 expression between diabetic and control mice, although expression tended to be lower in the DM2 group. However, Western blot analysis revealed a dramatically decreased expression of total Cx43 in the renal tissue of diabetic mice compared to the control group. Western blot, compared to immunohistochemical analysis, is a highly sensitive technique, while immunohistochemistry provides spatial distribution and is less sensitive to protein concentration. Hence, Western blot provides quantitative protein data because it generates a signal proportional to the total amount of protein in a sample. In contrast, IHC is often considered semi-quantitative and may not detect subtle overall reductions, although the trend was similar. The expression of the activated form, pCx43, also did not differ between groups when the total amount was measured. However, we noticed accumulation of pCx43 in cells of the vascular wall that were not endothelial. When pCx43 was colocalized with PDGFRB, we confirmed the assumption that more activated Cx43 is present in pericytes, and a slightly increased pCx43/PDGFRB expression was recorded in the kidneys of diabetic mice. The results on Cx43 expression in our diabetic model are only partially in agreement with previous studies. In the kidneys of diabetic mice, decreased expression of Cx43 was previously observed in the endothelium of the efferent arteriole, which was associated with an increase in intraglomerular capillary pressure and capillary damage [9,27]. In glomerular mesangial cells, high glucose concentration reduces Cx43 expression, which is associated with concomitant hypertrophy and ageing of these cells [28]. According to current data, in early DKD, high glucose triggers mesangial cell hypertrophy rather than immediate loss. This process is mechanistically linked to the downregulation of Cx43 and the subsequent impairment of gap junctional intercellular communication [29]. Furthermore, a reduction in pCx43 may indicate a phenotypic shift in which mesangial cells acquire myofibroblast-like features, characterized by increased production of extracellular matrix proteins such as collagen I, collagen IV, and fibronectin. This shift is often driven by TGF-β1 signalling, which can create a feedback loop that further suppresses Cx43 [5]. In addition, reduced expression of Cx43 in cortical tubule cells was found in diabetic rats in the DM1 model [30]. In our previous study on the DM1 model, we also found a decrease in cortical expression of Cx40 and Cx43 in diabetic rats [31]. However, in the rat model of DM2, a disruption of intercellular communication at the level of the juxtaglomerular apparatus (JGA) was found, with increased phosphorylation of Cx43 and decreased production of Cx40, Cx43, and Cx37 in renin-secreting cells [32]. The significance of altered Cx43 phosphorylation lies in its role as a functional switch between gap junctional intercellular communication and hemichannel activity. Elevated pCx43 is usually associated with the closure of gap junctions, preventing the homeostatic exchange of ions and nutrients between adjacent mesangial cells or pericytes. When gap junctions are inhibited, certain phosphorylation patterns can promote the pathological opening of undocked hemichannels [33]. Based on published data, open Cx43 hemichannels and PANX1 channels drive pro-inflammatory and pro-fibrotic signalling by facilitating ATP efflux [34]. Moreover, in agreement with our results, Cx43 expression was downregulated in DKD in db/db mice, a genetic model of DM2 [35].

Immunohistochemical expression of Cx43 in sections from human subjects partially colocalized with markers for collecting duct epithelium, aquaporin 2 (AQP2), synaptopodin—a marker for podocytes, PDGFRB—a marker for pericytes and mesangial cells, and CD31—a marker for endothelial cells. Using immunohistochemistry, we did not find a significant difference in total renal Cx43 expression between diabetic and control patients, nor in the expression of Cx43 in endothelial glomeruli, podocytes, mesangium, proximal tubules, or collecting ducts. We also did not find significant differences between groups in the total amount of activated pCx43. However, a highly and significantly decreased expression of pCx43 colocalized with PDGFRB (a marker of mesangial cells) was found in the kidneys of diabetic patients. Moreover, a negative correlation was found between the expression of pCx43 in the mesangium and the percentage of sclerotic glomeruli, which was used as a measure of the magnitude of renal damage. The role of connexins in the pathogenesis of diabetes mellitus and its complications has been intensively studied in numerous cell types, including pancreatic beta cells, endothelial and smooth muscle cells of blood vessels, and hepatocytes [10,36]. Under pathological conditions, intercellular communication is disrupted, which is also reflected in the changes in the expression, localization, or function of connexins [10]. Hyperglycemia leads to decreased communication between endothelial and smooth muscle cells in blood vessels [14,36]. Data on changes in connexin expression in the diabetic kidney are controversial. In renal biopsies from patients with type 2 diabetes mellitus, a decreased expression of Cx43 in podocytes was observed, which was associated with worsening diabetic kidney disease; that is, a decrease in renal function [16]. In another study, increased Cx43 expression was found in biopsy material from individuals with diabetic kidney disease, followed by decreased intercellular gap junctions and increased ATP release through the hemichannels [37]. Similar findings were also observed in injured human glomeruli from biopsies of patients with chronic kidney disease (CKD) and in the nephrotoxic glomerulonephritis murine model of CKD [14]. These data are only partially consistent with our results. The results from animal studies are also not uniform. High glucose concentration decreases the expression of Cx43 in rat microvascular endothelial cells, leading to reduced gap junction activity and disruption of intercellular communication [38]. Pharmacological or genetic blockade of Cx43 reduces proteinuria, blood urea nitrogen (BUN), and serum creatinine levels in the NT-GN mouse model [39] and in STZ-induced diabetes mellitus in rats [40]. However, overexpression of Cx43 attenuated renal fibrosis and reduced epithelial-to-mesenchymal transition in db/db mice and in rat kidney NRK-52E cells treated with high glucose [35]. A possible explanation for these discrepancies is that the expression and activation of Cx43 are not equally disturbed in different cellular populations, which we demonstrated in our present study. Opposite trends observed in mice versus human mesangial/pericyte populations probably reflect a transition from an acute compensatory phase (mouse) to a chronic degenerative phase (human), where different kinases dominate the Cx43 phosphorylation profile.

Pannexin channels are also associated with the pathogenesis of DM, but their role is much less studied. Increasing evidence highlights the importance of cell-to-cell communication via paracrine ATP in the pathophysiology of DKD and the central role of pannexins in ATP-mediated signalling [7,37,41,42,43,44]. Expression of PANX1 in pancreatic beta cells and adipocytes has been shown to be an important factor in the development of insulin resistance [45,46]. Panx2 is also present in pancreatic beta cells, and its deficiency in mice contributes to beta cell destruction and the development of insulin resistance [47]. Few studies have examined the expression or role of pannexin in the diabetic kidney [18,31,48,49]. However, data have shown that genetic deletion of PANX1, as well as pharmacological blocking of PANX1 channels with probenecid in mice, protects against acute kidney injury (AKI) caused by sepsis or ischemia/reperfusion [21,50]. In addition, it was found that tubular epithelial and endothelial PANX1 channels mediate AKI [51,52]. Therefore, tubular and endothelial PANX1 are a potential target for the treatment of various renal diseases.

Immunohistochemical expression of PANX1 in our study did not differ between diabetic and control mice. These results do not agree with our previous study on rats, where we found an increase in PANX1 expression in the kidneys of diabetic rats with streptozotocin-induced DM1 [31]. In that study, we observed the greatest increase in PANX1 in the distal tubules, which were the most damaged structures in all our previous studies using DM1 [24,31,53], but not in DM2 models [48,54]. We compared PANX1 expression in different renal cell populations of diabetic and non-diabetic patients. Immunohistochemical expression of PANX1 partially colocalized with markers for the collecting duct epithelium, aquaporin 2, nephrin—a marker for podocytes, CD31—a marker for endothelial cells, and PDGFRB—a marker for pericytes and mesangial cells. These results are consistent with our previous data on the distribution of PANX1 in different cell populations in the postnatal human kidney [18]. We also observed similar data using transmission electron microscopy in the rat kidney (in publication) and in our study of connexin and pannexin expression in mouse kidney [55].

We did not observe a significant difference in total PANX1 expression, or in PANX1 expression in glomeruli, podocytes, proximal tubules, or the collecting duct epithelium between diabetic and control patients.

However, PANX1 expression in CD31-immunoreactive endothelium was decreased in diabetic patients compared to control patients. Moreover, a negative correlation was found between the expression of PANX1 in glomerular CD31-immunoreactive endothelium and the percentage of sclerotic glomeruli, as a measure of the magnitude of renal damage. The action of PANX1 in endothelial cells involves several mechanisms. PANX1 mediates ATP release and subsequent paracrine purinergic signalling in smooth muscle cells and white blood cells. PANX1 in endothelial cells affects vascular permeability and facilitates the diapedesis of phagocytic leukocytes [8,56,57]. In addition, PANX1 regulates inflammatory signalling by facilitating the influx of extracellular Ca^2+^ into the endothelial cell cytoplasm. PANX1 channels are also involved in the modulation of vasoconstriction and vasodilation, consequently affecting blood pressure and glomerular filtration rate [8,56]. PANX1-mediated ATP release typically stimulates P2Y receptors on adjacent smooth muscle cells (SMCs) or acts in an autocrine manner to facilitate nitric oxide (NO) production. A reduction in endothelial PANX1 under diabetic conditions likely worsens the existing NO bioavailability deficit, further compromising the endothelium’s ability to maintain a healthy vasodilatory state [58].

One of the limitations of our study is that we used histologically ‘normal’ tissue adjacent to renal carcinoma, which might introduce potential confounding variables. However, DKD is rarely confirmed by needle biopsy, and the diagnosis is usually based on the presence of diabetes mellitus, as a primary disease together with signs of renal disease. On the other hand, concerning the control group, renal biopsy is not indicated in healthy people. Hence, the same way to obtain the tissue became a common research practice in studies of diabetic kidney disease [37,59]. There are very rare situations in which renal biopsy of healthy people is done and the material from autopsy is not appropriate due to intense autolysis. Large public studies (like The Cancer Genome Atlas Program), collected the specimens and data about the sections of the pathologically unchanged tissues obtained during the tumour extraction surgery. Although this is not ideal, it is widely used, because it would not be ethical to collect kidney samples surgically from healthy individuals.

A recent study by Lucero and collaborators [60] found that angiotensin II activates PANX1 channels on mesangial cells, leading to increased Ca^2+^ and ATP release. In addition, it was previously established that PANX1 also mediates angiotensin II-induced increases in mesangial cell permeability and pro-inflammatory cytokine release, contributing to the pathogenesis of CKD [22]. In agreement with these findings, the increased PANX1 expression we observed in the PDGFRB-immunoreactive mesangium of diabetic patients could be one of the mechanisms involved in the pathogenesis of renal damage during diabetes.

The main drawback of our study is the relatively small sample. However, despite the small sample size, the main observed differences were significant, suggesting that the observed effect size was likely large. Therefore, we believe that our results contribute to our knowledge of the connexin and pannexin role in diabetic kidney disease. However, to determine the underlying mechanisms, prospective experimental studies are needed, including knock-out mouse models with the specific deletion of connexins/pannexins in distinct cell populations. However, we believe that the results of our present study provide good direction for future studies.

## 4. Materials and Methods

### 4.1. Type 2 Diabetes Model in Mice

The national Ethics Committee and the Ministry of Agriculture of the Republic of Croatia approved the study (Class: UP/I-322-01/23-01/23; Reg. no.: 525-09/589-23-4), as did the Ethics Committee of the University of Split School of Medicine (Class: 003-08/23-03/0015; Reg. no.: 2181-198-03-04-23-0063). Eight- to ten-week-old C57BL/6J mice (*n* = 14) were raised in the Animal Facility of the University of Split. Food (4RF25 CS; PF1609; Mucedola S.r.l., Settimo Milanese, Italy) and water were available ad libitum. Type 2 diabetes was induced in 9 mice (5 females and 4 males) according to Shi et al. [23] with a low dose of streptozotocin (STZ; 120 mg/kg in 0.9% NaCl i.p.), and 3 weeks after diabetes confirmation, the normal diet was replaced with a high-fat diet (HFD—60% of energy from fat; Altromin C1090-60 Purified high-fat diet; Altromin, Lage, Germany). Control animals (3 females and 2 males) received a carrier (0.9% NaCl, i.p.) and were fed a control diet (C 1090 - 10C Purified control diet, 10% kilocalories from fat; Altromin, Lage, Germany) until the end of the experiment. Animals were euthanized 12 weeks after the start of the HFD (total 16 weeks in the experiment; 24–26 weeks old). Before STZ administration, blood glucose (b.g.) was measured in all animals using a glucometer (Accu Check Instant, Roche, Indianapolis, IN, USA) from a drop of blood taken from the tail vein, and body weight was measured. Body weight and b.g. levels were measured by the same procedure once every 2 weeks.

At the end of the experiment, animals were anesthetized with isoflurane and euthanized by cervical dislocation. Urine was collected directly, and the concentration of albumin, total proteins, creatinine, and glucose in the urine was determined (Combina 13, COMBN0001, Biotek Solutions, Skopje, North Macedonia). A portion of tissue from one kidney was fixed in 4% buffered formalin for histological and immunohistochemical (IHC) analyses, while another portion was stored in McDowell fixative for TEM, and a third portion was snap-frozen in liquid nitrogen and stored at −80 °C for protein isolation. The kidney tissue was embedded in paraffin, cut into 5 μm thick histological sections, and stained using standard histological techniques (hematoxylin-eosin, Mallory’s trichrome staining, and Sirius red for the detection of fibrotic changes, and Periodic acid–Schiff—PAS for the detection of glycogen accumulation). Changes were observed and photographed using a light microscope and documented with a digital camera (BX40, Olympus, Tokyo, Japan) and a camera (DP27, Olympus, Tokyo, Japan). Pathological changes including the degree of tubulointerstitial fibrosis, glomerular sclerosis, and tubular vacuolization, were analyzed by two independent pathologists and quantified using the ImageJ programme (version 1.48, NIH, Bethesda, MD, USA).

### 4.2. Human Tissue Procurement and Processing

We analyzed renal tissue from 20 patients who underwent nephrectomy for renal carcinoma at the University Hospital of Split (Table S1): 9 patients with type 2 diabetes mellitus 2 (DM2) and 11 patients without a diabetes diagnosis. The Ethics Committee of the University of Split School of Medicine approved the study (Class: 003-08/23-03/0015; Reg. no.: 2181-198-03-04-23-0063). Normal tissue adjacent to the carcinoma was fixed in buffered 4% paraformaldehyde. Laboratory data at the time of nephrectomy were obtained from hospital records.

### 4.3. Immunohistochemistry Procedure

Human and mouse renal tissue samples were dehydrated in ethanol, cleared in xylene, and embedded in paraffin wax following a standard procedure [61]. Paraffin blocks were cut into 5 μm thick sections and mounted on glass slides. Sections were then deparaffinized in xylene, rehydrated in decreasing concentrations of ethanol in water, and finally rinsed with distilled water. Antigen retrieval was performed by heating in sodium citrate buffer (pH 6.0) for 30 min, using a steam cooker. After cooling to room temperature, sections were washed in PBS. Protein block (ab64226, Abcam, Cambridge, UK) was applied to prevent nonspecific antibody binding and incubated for 20 min. After removing the protein block, a combination of primary antibodies diluted in PBS (Table 1) was applied, and incubation with primary antibodies in a humid chamber lasted overnight. Sections were then washed in PBS, and the appropriate combination of secondary antibodies was applied and incubated for 1 h in a humid chamber (Table 1). After washing in PBS, nuclei were stained with 4′,6′-diamidino-2-phenylindole dihydrochloride (DAPI), washed with distilled water, and the slides were coverslipped (Immumount, Shandon, Pittsburgh, PA, USA). Omission of the primary antibody from the procedure resulted in no tissue staining.

### 4.4. Data Acquisition and Analysis

Sections were viewed using a BX51 microscope (Olympus, Tokyo, Japan) and captured using a cooled digital camera (DS-Ri2; Nikon, Tokyo, Japan) with NIS-Elements F software (version 4.60; Nikon, Tokyo, Japan). To quantify immunoexpression of connexin/pannexin in different cellular populations of diabetic and nondiabetic patients’ kidneys, six visual fields were captured at 40× objective magnification with a constant exposure time for each filter (green or red). Non-overlapping randomly selected visual fields were photographed for the subsequent quantitative immunofluorescence analysis.

Photomicrographs were processed and analyzed using ImageJ software (version 1.48, National Institutes of Health, Bethesda, MD, USA). The red counter signal was subtracted from the images with green. A median filter with a radius of 2.0 pixels was then applied, and the threshold was adjusted using the “Triangle” thresholding algorithm. The percentage area under the double-fluorescence was determined using Adobe Photoshop (Adobe Inc., San Jose, CA, USA). To analyze glomeruli, specific structures were manually outlined and isolated in Adobe Photoshop (Adobe Inc., San Jose, CA, USA). The number of pixels in the overlapping (yellow) area and the cell-marker area (red) was measured, and the percentage of overlapping area was calculated as previously described [62]. Image acquisition and quantitative analysis were performed in a blinded manner. For presentation purposes, subtraction of background and slight contrast adjustment were performed.

### 4.5. Kidney Tissue Protein Extraction

Tissues intended for protein isolation were homogenized in 1000 µL of RIPA buffer (pH 7.4; 50 mM Tris-HCl, 1 mM EDTA, 150 mM NaCl, 1% Triton X-100, 0.5% sodium deoxycholate, 0.1% SDS) supplemented with a protease inhibitor cocktail, using a Precellys Minilys homogenizer (two cycles of 30 s at 5000 rpm, with a 10-second cooling period on ice between cycles). After homogenization, samples were centrifuged for 10 min at 10,000 rpm at 4 °C. The protein-containing supernatant was transferred to a new microcentrifuge tube and incubated on a rocking platform at 4 °C for 2 h. This was followed by a final centrifugation for 15 min at 10,000 rpm at 4 °C. The total protein lysate was transferred to a new microcentrifuge tube, mixed with 4× Laemmli buffer, and denatured at 95 °C for 5 min. The prepared samples were then ready for Western blot analysis.

### 4.6. Western Blotting

An aliquot of 25 µL protein lysate was loaded onto a 12% polyacrylamide gel for SDS-PAGE, followed by transfer to a polyvinylidene fluoride (PVDF) membrane (Merck-Millipore) using a wet transfer system (BioRad, Hercules, CA, USA). The membranes were blocked by shaking for 1 h in 5% milk diluted in TBS-Tween (20 mM Tris, 150 mM NaCl, 0.05% Tween 20). The membrane was then incubated overnight at 4 °C with a primary antibody diluted in 5% BSA in TBS-Tween, washed three times in TBS-T, and incubated for 2 h with the secondary antibody diluted in TBS-Tween. The proteins of interest were detected by either chemiluminescence or fluorescence. For chemiluminescence, SuperSignal Pico Substrate was used, and X-ray films (FUJI FPM 100A, Fujifilm, Tokyo, Japan) were exposed to the membranes and developed in a Fuji Medical Film Processor (FPM-100A, Fuji Photo Film Co., Ltd., Tokyo, Japan). For fluorescence, the Typhoon™ FLA 9500 biomolecular imager (GE Healthcare Life Sciences, Chicago, IL, USA) was used, and the images were analyzed using ImageJ (Fiji, Tokyo, Japan).

### 4.7. Transmission Electron Microscopy

Pieces of renal tissue from the left kidney were immersed in McDowell fixative and processed for resin embedding, as described previously [24]. The following day, sections were post-fixed in 1% osmium tetroxide for 1 h and then dehydrated in acetone and embedded in Spurr resin (Sigma-Aldrich Inc., St. Louis, MO, USA). The sections were examined with a transmission electron microscope (JEM JEOL 1400, Jeol Ltd., Tokyo, Japan).

### 4.8. Statistical Analysis

All statistical tests were performed using GraphPad Prism (GraphPad Software, version 8.0.1 for Windows, San Diego, CA, USA). A priori power calculation was not done. Data were expressed as mean values ± standard deviation (SD). The *t*-test for unequal variances was used to compare differences between patient groups. Pearson’s correlation coefficient was calculated between the expression of connexin or pannexin in different populations and the percentage of sclerotic glomeruli. A *p*-value less than 0.05 was considered significant.

## 5. Conclusions

In conclusion, the expression and activation of Cx43, as well as the expression of PANX1, are altered in specific populations of renal cells during long-term type 2 diabetes mellitus, particularly in vascular wall cells. This may suggest their involvement in the pathophysiological processes underlying diabetic kidney development, which should be explored further in future studies. The results of this study enhance our understanding of the roles of connexin and pannexin in intercellular communication among different cell types during the development of diabetic kidney disease. This knowledge is essential for the targeted and effective modulation of connexin and pannexin channels as therapeutic strategies for the prevention and treatment of diabetic kidney disease.

## Figures and Tables

**Table 1:** List of primary and secondary antibodies used.

	Antibody	Code No.	Host	Dilution	Source
Primary	Anti-pannexin 1/PANX1	ABN242	Rabbit	1:300	Merck KGaA, Darmstadt, Germany
	Anti-Connexin 43 Antibody (F-7)	sc-271837	Mouse	1:50 (IHC) 1:1000 (WB)	Santa Cruz Biotechnology Inc., Santa Cruz, CA, USA
	Anti-Connexin 43/GJA1 antibody-C-terminal	ab219493	Goat	1:300	Abcam, Cambridge, UK
	Anti-phospho-Connexin 43 (pSer368)	SAB4504371	Rabbit	1:100	Sigma-Aldrich, St. Louis, MO, USA
	Anti-nephrin (B-12)	sc-377246	Mouse	1:50	Santa Cruz Biotechnology Inc., Santa Cruz, CA, USA
	Anti-Podocin/NPHS2 Antibody (JB51-33)	NBP2-75624	Rabbit	1:100	Novus Biologicals, Centennial, CO, USA
	Anti-CD31/PECAM-1 Antibody	NB100-2284	Rabbit	1:100	Novus Biologicals, Centennial, CO, USA
	Anti-PECAM-1 Antibody	Sc-53389	Mouse	1:50	Santa Cruz Biotechnology Inc., Santa Cruz, CA, USA
	Anti-PDGFRβ	AF386	Goat	1:20	R&D Systems, Inc., McKinley Place NE, MN, USA
	Anti-Aquaporin 2/AQP2 (E-2)	sc-515770	Mouse	1:50	Santa Cruz Biotechnology Inc., Santa Cruz, CA, USA
	Anti iba1	ab153696	Rabbit	1:100	Abcam, Cambridge, UK
	Anti-CD3	C7930	Rabbit	1:200	Sigma-Aldrich, St. Louis, MO, USA
	Anti pannexin 1	FAB7097	Mouse	1:1000	Abcam, Cambridge, UK
	Anti b-Actin (C4)	sc-47778	Mouse	1:1000	Santa Cruz Biotechnology Inc., Santa Cruz, CA, USA
	TNF alpha Antibody (52B83)	sc-52746	Mouse	1:1000	Santa Cruz Biotechnology Inc., Santa Cruz, CA, USA
	TGF beta 1 Antibody (3C11)	sc-130348	Mouse	1:1000	Santa Cruz Biotechnology Inc., Santa Cruz, CA, USA
	Anti-Collagen I	SAB4500362	Rabbit	1:1000	Sigma-Aldrich, St. Louis, MO, USA
	Anti-GAPDH, clone 14C10	2118S	Rabbit	1:1000	Cell Signalling Technology, Danvers, MA, USA
Secondary	Alexa Fluor^®^488 AffiniPure Anti-Rabbit lgG (H+L)	711-545-152	Donkey	1:300	Jackson Immuno Research Laboratories, Inc., Baltimore, PA, USA
	Rhodamine Red™-X (RRX) AffiniPure Anti-Mouse IgG (H+L)	715-295-151	Donkey	1:300	Jackson Immuno Research Laboratories, Inc., Baltimore, PA, USA
	Rhodamine Red™-X (RRX) AffiniPure Donkey Anti-Rabbit IgG (H+L)	711-295-152	Donkey	1:300	Jackson Immuno Research Laboratories, Inc., Baltimore, PA, USA
	Rhodamine Red™-X (RRX) AffiniPure donkey anti-goat IgG (H+L)	705-295-003	Donkey	1:300	Jackson Immuno Research Laboratories, Inc., Baltimore, PA, USA
	Goat Anti-Mouse IgG H&L (HRP)	AB205719	Goat	1:2000	Abcam, Cambridge, UK
	Goat anti-rabbit IgG HRP-linked	#7074	Goat	1:2000	Cell Signalling Technology, Danvers, MA, USA

## Data Availability

The original contributions presented in this study are included in the article. Further inquiries may be directed to the corresponding author.

## Supplementary Materials

The following supporting information can be downloaded at: https://www.mdpi.com/article/10.3390/ijms27052152/s1.
