# Supplementary material for: Connexin 43 and Pannexin 1 in Renal Cell Populations in Diabetic Kidney Disease
Source: Int J Mol Sci. 2026 Feb 25;27(5):2152. doi: 10.3390/ijms27052152 (PMC12984592; doi:10.3390/ijms27052152)
Supplement: Supplementary file 1 [file ijms-27-02152-s001.zip › ijms-4123657-supplementary.pdf]

Supplementary table 1. Characteristics of patients in the study

	**Control group**	**Diabetic group**
Age (years)	66.27 ± 8.83	65.44 ± 6.89
Sex (F/M)	3 / 8	3 / 6
Serum creatinine (µmol/l)	91.27 ± 22.12	104.22 ± 46.07
Sclerotic glomeruli (%)	9.78 ± 5.16	35.98 ± 134.14
